# Evaluation of Elements Spine SRS Plan Quality for SRS and SBRT Treatment of Spine Metastases

**DOI:** 10.3389/fonc.2020.00346

**Published:** 2020-04-03

**Authors:** Michael Trager, Angelia Landers, Yan Yu, Wenyin Shi, Haisong Liu

**Affiliations:** Department of Radiation Oncology, Sidney Kimmel Medical College, Thomas Jefferson University, Philadelphia, PA, United States

**Keywords:** spine SRS, spine SBRT, spine radiosurgery, spine metastases, plan quality evaluation

## Abstract

**Purpose:** The Elements Spine Stereotactic Radiosurgery treatment planning system uses automated volumetric modulated arc radiotherapy that can provide a highly conformal dose distribution to targets, which can provide superior sparing of the spinal cord. This study compares the dosimetric quality of Elements plans with the clinical plans of 20 spine stereotactic radiosurgery/stereotactic body radiation therapy (SRS/SBRT) patients treated at our institution.

**Methods:** Twenty spine SRS/SBRT patients who were clinically treated at our institution were replanned using the automated Elements planning workflow with prespecified templates. Elements automatically evaluates the size and shape of the target to determine if splitting the PTV into simplistic subvolumes, each treated by their own arc(s), would increase conformity and spinal cord sparing. The conformity index, gradient index, PTV *D*_5%_, and maximum and mean cord dose were evaluated for the Elements and clinical plans. Treatment delivery efficiency was also analyzed by comparing the total number of monitor units and the modulation factor. Wilcoxon rank-sum tests were performed on the statistics.

**Results:** Elements split the PTV for 50% of cases, requiring four or six arcs. Overall, Elements plans were found to be superior to clinical plans in conformity index, gradient index, and maximum cord dose. The PTV *D*_5%_ and cord mean dose for the Elements plans trended higher and lower, respectively. The numbers of monitor units and modulation factor were also higher for Elements plans, although the differences were not significant.

**Conclusion:** Automated Elements plans achieved superior conformity and cord dose sparing compared to clinical plans and PTV splitting successfully improved spinal cord sparing.

## Introduction

Spine metastases are a common complication of various cancers that can cause severe pain and even lead to neurological problems (1). Although bone metastases are typically associated with poor prognosis, modern advances in systemic and supportive therapies continually improve patients' life expectancy (2). Treatment with external beam radiation therapy is a convenient and effective method to mitigating this pain (3). With advances in imaging and delivery techniques, stereotactic radiosurgery (SRS) and stereotactic body radiation therapy (SBRT) are being increasingly used to manage these spine metastases (4). Stereotactic radiosurgery and SBRT offer patients more rapid and durable pain relief than conventional therapy with the added benefit of a shorter course of treatment (5, 6). One major challenge to treating spine metastases is proximity to the spinal cord and extent of dural involvement (7). Vendors are now developing and releasing advanced treatment planning systems (TPSs) with tools that allow for complex sculpting of dose to create steep dose gradients near critical structures, such as the spinal cord, as well as implementing automation and artificial intelligence to increase planning throughput and consistency based on previous investigations (8, 9).

**Figure 1:**
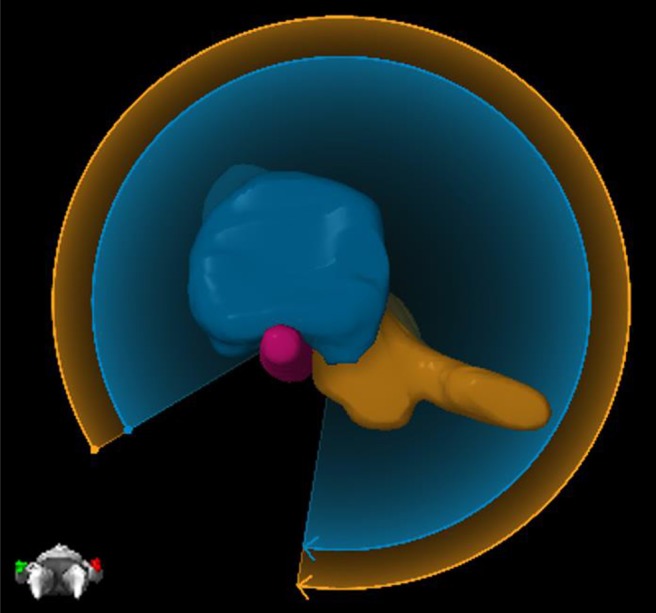
An example of PTV splitting. Because of the complexity of the target between the blue and orange partitioned areas, Elements will deliver two arcs where each arc focuses solely on one part of the target. The blue arc will deliver dose solely to the blue part of the target, and then the orange arc will deliver dose solely to the orange part of the target.

Elements Spine SRS (Brainlab AG, Munich, Germany; referred to as Elements moving forward) is a TPS that allows for a highly automated planning process specialized in obtaining a steep dose gradient at the target and spinal cord interface. Recurrence in the epidural space at the interface of the spinal cord is one of the primary mechanisms of failure after spine SRS and SBRT (10). The spinal cord interface is a challenging trade-off region for any generic volumetric modulated arc therapy (VMAT) algorithm. Elements, however, has a dedicated spine VMAT algorithm designed to deposit higher dose at the interface. Elements automatically partitions the target volume into a number of smaller targets, which are less complex in terms of concavity, and thereby allows the optimizer to create a steeper dose gradient with optimal sparing of the spinal cord (referred to as PTV splitting moving forward). Figure 1 depicts one example of PTV splitting. Because it would be hard to deliver a conformal dose to the complex concave region between the blue and orange partitions of the target if treated as one target, the blue arc will deliver dose solely to the blue part of the target, whereas the orange arc will deliver dose solely to the orange target.

Treatment planning for spine radiosurgery often demands significant experience and a large time commitment from treatment planners. Automation can provide consistent quality treatment plans regardless of anatomical complexity. The template-driven treatment planning in Elements requires organ at risk (OAR) constraints to be input only once prior to clinical implementation for each fractionation scheme used. Elements employs Monte Carlo (MC) optimization because the difference between dose distributions obtained with pencil-beam (PB) algorithms and MC can be significant for thoracic spine (interface between air and bone) and paraspinal (interface between soft tissue and bone) tumors, as well as regions with spinal implants (interface between metal and bone).

To achieve quick and accurate VMAT optimization, Elements utilizes two separate dose calculations in an intertwined manner. A beamlet-based approximate dose calculation is used for quick calculations, and a forward dose calculation is used for high-accuracy calculations. Most of the necessary calculations during optimization are performed with the quick beamlet-based calculation; however, the forward dose calculation is used for correcting the dose values of the approximate calculation with linear scaling. This hybrid beamlet approximation and forward dose calculation method allow for a quick and accurate dose calculation during optimization.

Few studies have been performed to directly compare the capabilities of various TPSs with respect to spine SRS and SBRT. Saenz et al. (11) demonstrated the dosimetric advantage of Elements' spine-specific optimization compared to Pinnacle (3) and Monaco, which use generalized optimizations. Nalichowski et al. (12) reported a thorough study on spine SRS/SBRT dosimetry and treatment efficiency with CyberKnife, Tomotherapy, Vero, and Eclipse/Truebeam. They found dosimetric advantages with Vero and CyberKnife and faster delivery times with Truebeam and Tomotherapy. This study aims to compare the plan quality and delivery efficiency of the Elements TPS with accepted clinical plans that were previously treated at our institution. These clinical plans were generated with Eclipse TPS (Varian Medical Systems, Palo Alto, CA, USA).

## Methods

### Treatment Planning

Twenty clinically treated and physician-approved spine SRS and SBRT (C-spine: *n* = 1, C/T-spine: *n* = 2, T-spine: *n* = 6, L-spine: *n* = 9, and sacrum: *n* = 1) patient plans created in Eclipse TPS were replanned using the Elements TPS and retrospectively analyzed. All clinical plans were created using RapidArc inverse optimization with two full rotation coplanar arcs. Optimization was based on physician-provided OAR constraints, following RTOG-0631 guideline. Dose calculations were performed on 2-mm dose grid using AAA algorithm, and heterogeneity correction was enabled. A wide range of prescriptions were included. See Table 1 for details on each case. For consistency, all plans were normalized such that 95% of the PTV received 100% of the prescription dose. Varian Truebeam linac (equipped with 5-mm Millennium™ 120-leaf MLC), 6MV photon beam was used [regular mode: *n* = 16, and flattening filter free (FFF) mode: *n* = 4].

**Table 1:** Summary of all cases analyzed.

**Case number**	**Treatment site**	**Target volume (cc)**	**Prescription dose (Gy)**	**Fractions**	**Energy (MV)**
1	T5	14.98	18	1	6
2	L1	39.30	16	1	6
3	L2	62.57	16	1	6
4	S	78.47	14	1	6
5	T11	62.82	24	1	6FFF
6	C7–T1	79.35	30	5	6
7	L2	45.50	24	3	6
8	T9	31.10	24	3	6FFF
9	T8	60.72	30	5	6
10	C4	37.63	30	5	6
11	L3	105.96	16	1	6
12	L3	45.82	16	1	6
13	L4–5	147.24	18	1	6FFF
14	T2–T5	107.18	12	1	6
15	L4	46.47	27	3	6
16	T11	79.25	27	3	6
17	T2–T5	31.53	24	3	6
18	C4–T1	81.96	24	3	6
19	L3	81.24	24	3	6
20	L5	50.50	18	1	6FFF

Same linac and same energy (mode) were used during replan. Elements utilizes an adaptive dose grid based on structure size and interface regions between the target and critical structures. For an average-sized PTV in Elements from our 20 cases of 64.48 ± 31.51 cc, the dose grid size was 2 mm, and for a typical-sized spinal cord the dose grid size was 1.8 mm. Elements utilizes a pencil beam algorithm for initial optimization and dose calculation and then allows for an optional final MC optimization and dose calculation. All plans in this study were analyzed after the final MC dose calculations. Monte Carlo calculation is always performed with an isomorphic dose grid size of 2 mm.

For VMAT planning in general, if it is only possible to set minimum or maximum MU values, it may not guarantee absence of some complex apertures in the sequence. Elements, however, allows the user to directly control the amount of modulation by using a modulation control in the software. The highest possible modulation was allowed to give the optimizer more freedom. In Elements Spine SRS, the arc configuration setup is obtained through user-predefined template. In this study, the arc setup template is taken from our prior experienced setup we had with RapidArc, which is the two full-rotation coplanar arcs. However, Elements may duplicate the arc setup, based on the software's analysis of the PTV size and shape and its PTV splitting functionality, to make the final plans with either four arcs (PTV split once) or six arcs (PTV split twice).

The prespecified template accounts for most treatment planning parameters. Templates in this study were created with consideration for only PTV coverage and sparing of the maximum dose to the spinal cord (or cauda equine). PTV coverage is normalized so that 95% of the volume is covered by the prescription dose. For spinal cord, the maximum point dose (defined as ≤ 0.035 cc) is set to be <14, 22.5, and 28 Gy for one, three, and five fractions, respectively, in planning templates; 0.35-cc volume of spinal cord dose is set to be <10, 16, and 22 Gy for one, three, and five fractions, respectively. For cauda equine, the maximum point dose is set to be <16, 24, and 32 Gy for one, three, and five fractions, respectively; 5-cc volume of cauda equine dose is set to be <14, 22, and 30 Gy for one, three, and five fractions, respectively. In actual clinical case planning, users can easily turn on other OARs (lung, liver, stomach, etc.) with user-defined constraints in template.

The study was approved by the office of human research, institutional review board (IRB) of Thomas Jefferson University. The IRB has granted a waiver of informed consent.

### Dosimetric Analysis

Parameters used for dosimetric plan evaluation were conformity index (CI), gradient index (GI), *D*_5%_ to the PTV, *D*_max_ to the cord, and *D*_mean_ to the cord. The CI was defined as

$$\begin{array}{l} CI=\frac{V_{RX}}{V_{PTV}⋂V_{RX}}\frac{V_{PTV}}{V_{PTV}⋂V_{RX}} \end{array}$$

where *V*_RX_ is the volume enclosing the prescription isodose line (IDL), and *V*_PTV_ is the volume of the PTV. The GI was defined as

$$\begin{array}{l} GI=\frac{V_{50\%}}{V_{PTV}} \end{array}$$

where V_50%_ is the volume enclosed by the 50% IDL. Max dose to the cord is defined by the dose to 0.03 cc of the cord.

The CI and GI were obtained with an independent calculation to ensure consistency between planning systems using relevant values directly extracted from the dose volume histograms (DVHs) of each TPS. The *D*_5%_ to the PTV and *D*_max_ to the cord were obtained directly from the DVH curves. The *D*_mean_ to the cord was reported as the value displayed in each TPS. A Wilcoxon rank-sum test performed in MATLAB 9.2 (MathWorks, Natick, MA, USA) was used to determine significance of the findings.

### Efficiency Analysis

Treatment delivery efficiency was compared between both planning systems with total monitor units (MUs) and a modulation factor (MF), which is defined as the total MUs for all fractions divided by the total dose in Gray. A Wilcoxon rank-sum test performed in MATLAB 9.2 was used to determine significance of the findings.

## Results

### Overall Results

**Figure 2:**
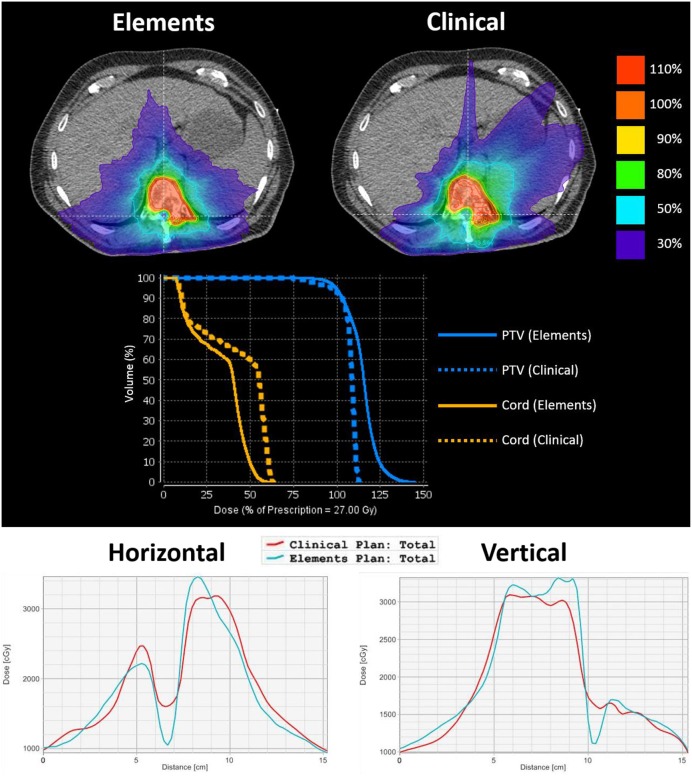
An axial image from case 16's plans to emphasize differences in dose gradient at the spinal cord–target interface. The target volume is delineated by a yellow contour. The top left shows IDLs from the Elements plan, and the top right shows IDLs for the clinical plan. The middle shows the corresponding DVH curves of PTV and spinal cord curves for each plan. The bottom left shows the 2D dose profile along a horizontal line crossing through the cord, and the bottom right shows the 2D dose profile along a vertical line crossing through PTV and spinal cord (lines were shown in the above isodose picture overlaid on axial CT image). From both 2D dose profiles, it can be seen that Elements plan achieved lower cord dose and faster dose fall off between PTV and cord.

One of the main advantages noticed in the Elements plans was a steeper dose gradient at the spinal cord–target interface. This steeper gradient allows for superior target coverage in close proximity to the spinal cord, where there is risk of recurrence, while simultaneously decreasing the maximum dose to the spinal cord. The difference in dose gradient is evident in Figure 2, which is an axial slice and DVH from case 16's plans. The target volume is delineated by the yellow contour. The top left shows IDLs from the Elements plan, whereas the top right shows IDLs for the clinical plan. The prescription IDLs in the Elements plan cover more of the target in close proximity to the spinal cord than the clinical plan while also creating a ring of the 50% IDL (13.5 Gy) around the spinal cord where the same IDLs in the clinical plan completely engulf the spinal cord. These results are also shown by the DVH curves in the middle section of Figure 2, and two-dimensional (2D) dose profiles plotted along patient's right-to-left direction (bottom left in Figure 2) and anterior to posterior direction (bottom right in Figure 2) crossing the middle of spinal cord on this axial CT image.

A summary of results for all dosimetric parameters can be found in Table 2. Conformity index is 1.10 ± 0.04 for Elements plans and 1.25 ± 0.16 for clinical plans. Gradient index is 3.49 ± 0.32 for Elements plans and 4.21 ± 0.74 for clinical plans. It can be seen that Elements plans achieved not only better conformity and dose fall off (less in the mean values of CI and GI), but also more consistent quality plans as well (less in the standard deviation of CI and GI).

**Table 2:** A summary of results for all dosimetric parameters analyzed including means and standard deviations (SDs) for conformity and gradient indices.

**Case number**	**CI**		**GI**		**PTV** ***D***_****5%****_ **(Gy)**		**Cord** ***D***_****max****_ **(Gy)**		**Cord** ***D***_****mean****_ **(Gy)**	
	**Elements**	**Clinical**	**Elements**	**Clinical**	**Elements**	**Clinical**	**Elements**	**Clinical**	**Elements**	**Clinical**
1	1.10	1.24	3.29	3.74	23.44	20.95	6.12	9.45	3.50	6.10
2	1.08	1.23	3.37	3.55	20.44	18.50	6.14	8.78	0.48	0.69
3	1.08	1.25	3.09	3.71	20.65	19.16	11.41	14.63	0.21	3.15
4	1.11	1.11	3.03	4.12	18.15	14.95	10.32	13.08	1.86	3.03
5	1.09	1.18	3.19	3.77	31.08	27.66	8.69	9.90	1.71	1.95
6	1.11	1.11	3.26	3.55	40.70	35.68	15.09	18.42	7.30	10.6
7	1.10	1.52	3.38	3.61	30.67	30.02	10.39	13.18	3.83	5.60
8	1.10	1.15	3.19	3.75	31.17	26.28	13.44	17.36	1.61	2.43
9	1.15	1.38	3.40	3.97	40.74	34.32	12.18	14.44	8.63	11.34
10	1.08	1.20	3.27	3.83	38.84	32.74	19.50	23.14	6.27	6.45
11	1.09	1.11	3.60	4.29	20.32	16.69	11.31	14.63	4.21	6.53
12	1.09	1.12	3.99	3.96	20.15	17.24	12.19	15.42	3.33	4.46
13	1.12	1.20	3.44	6.21	22.63	19.74	9.72	12.20	3.84	4.71
14	1.06	1.71	3.91	4.63	15.08	19.83	7.56	10.68	4.15	5.71
15	1.12	1.11	4.08	5.25	32.29	29.67	18.82	22.39	3.94	5.08
16	1.11	1.40	3.90	4.37	34.83	31.24	13.91	17.49	8.72	12.19
17	1.11	1.26	3.50	3.96	31.71	27.23	9.22	11.04	6.77	8.11
18	1.20	1.29	3.41	4.01	31.41	27.23	5.64	7.21	3.40	4.10
19	0.99	1.14	4.02	5.87	29.73	26.45	19.55	23.10	7.73	10.04
20	1.12	1.36	3.40	4.11	23.17	20.49	9.27	15.76	4.10	7.25
Average	1.10	1.25	3.49	4.21						
SD	0.04	0.16	0.32	0.74						

*Mean and SD were not given for the statistics of these columns because differences in prescriptions would heavily influence the reported values. Therefore, these spaces are grayed out*.

The same information can be found in the form of average ratios of Elements plan to clinical plan values in Table 3 in order to normalize differences in prescription dose and individual treatment scenario. Values >1 indicate a larger value for the parameter in the Elements, and values <1 indicate the opposite. The Elements plans were significantly more conformal than current clinical plans in 17/20 instances with an overall average CI difference of −0.15 ± 0.16 (range, −0.65 to +0.01; *p* = 2.30e-05) and equivalent in 2/20 plans. The GI for the Elements plans was significantly lower than current clinical plans in 19/20 instances with an overall average GI difference of −0.73 ± 0.62 (range, −2.77 to +0.03; *p* = 7.41e-05). The maximum dose to 5% of the PTV was higher in 19/20 of the Elements plans with an average difference of +3.06 ± 2.33 Gy (range, −4.75 to +6.42 Gy; *p* = 0.16). The maximum dose to the cord was significantly lower in all Elements plans with an average difference of −3.09 ± 1.08 Gy (range, −3.92 to −1.21 Gy; *p* = 0.036), and the mean dose trended lower in all Elements plans with an overall average difference of −1.60 ± 1.05 Gy (range, −3.47 to −0.18 Gy; *p* = 0.099).

**Table 3:** Ratios (elements values to clinical values) of dosimetric parameters analyzed.

**Case number**	**CI**	**GI**	**PTV *D*_**5%**_**	**Cord *D*_**max**_**	**Cord *D*_**mean**_**
1	0.88	0.88	1.12	0.65	0.57
2	0.88	0.95	1.10	0.70	0.70
3	0.87	0.83	1.08	0.78	0.66
4	1.00	0.74	1.21	0.79	0.61
5	0.92	0.85	1.12	0.88	0.88
6	1.00	0.92	1.14	0.82	0.69
7	0.72	0.94	1.02	0.79	0.68
8	0.95	0.85	1.19	0.77	0.66
9	0.83	0.86	1.19	0.84	0.76
10	0.90	0.85	1.19	0.84	0.97
11	0.98	0.84	1.22	0.77	0.64
12	0.98	1.01	1.17	0.79	0.75
13	0.93	0.55	1.15	0.80	0.82
14	0.62	0.84	0.76	0.71	0.73
15	1.01	0.78	1.09	0.84	0.78
16	0.79	0.89	1.11	0.80	0.72
17	0.88	0.88	1.16	0.84	0.83
18	0.93	0.85	1.15	0.78	0.83
19	0.87	0.68	1.12	0.85	0.77
20	0.82	0.83	1.13	0.59	0.57
Average	0.89 ± 0.10	0.84 ± 0.10	1.12 ± 0.10	0.78 ± 0.07	0.73 ± 0.10

*Ratios >1 indicate a larger value in the Elements software. Ratios <1 indicate the opposite*.

Results for all case-specific planning efficiency parameters can be seen in Table 4. Elements had more MUs and MFs than current clinical plans in 12/20 instances and an overall average MU increase of 547.80 ± 1,599.56 (*p* = 0.62) and MF increase of 0.32 ± 0.71 (*p* = 0.30); however, neither was significant. PTV splitting was not utilized by Elements for cases 1–10.

**Table 4:** A summary of results from the efficiency analysis for all cases.

**Case number**	**Prescription dose/fx (#fxs)**	**MU**		**MF**		**PTV splitting? (arcs used; Elements)**
		**Elements**	**Clinical**	**Elements**	**Clinical**	
1	18 (1)	7,055	5,278	3.92	2.93	*n* (2)
2	16 (1)	5,799	4,674	3.62	2.92	*n* (2)
3	16 (1)	4,574	3,214	2.86	2.01	*n* (2)
4	14 (1)	3,382	3,859	2.42	2.76	*n* (2)
5	24 (1)	11,154	11,930	4.65	4.97	*n* (2)
6	6 (5)	8,110	8,295	2.70	2.77	*n* (2)
7	8 (3)	9,717	9,510	4.05	3.96	*n* (2)
8	8 (3)	8,022	8,130	3.34	3.39	*n* (2)
9	6 (5)	9,435	12,685	3.15	4.23	*n* (2)
10	6 (5)	7,175	7,740	2.39	2.58	*n* (2)
11	16 (1)	6,428	6,321	4.02	3.95	*y* (4)
12	16 (1)	6,163	4,918	3.85	3.07	*y* (6)
13	18 (1)	8,743	6,551	4.86	3.64	*y* (6)
14	12 (1)	4,532	4,713	3.78	3.93	*y* (6)
15	9 (3)	9,393	8,646	3.48	3.20	*y* (4)
16	9 (3)	10,269	11,850	3.80	4.39	*y* (4)
17	8 (3)	11,658	10,107	4.86	4.21	*y* (4)
18	8 (3)	8,469	7,371	3.53	3.07	*y* (4)
19	8 (3)	9,939	5,820	4.14	2.43	*y* (6)
20	18 (1)	9,255	6,704	5.14	3.72	*y* (6)

*Elements chose to use PTV splitting for cases 11–20 but not cases 1–10*.

### Results Stratified by the Number of Fractions

Results for mean values for CI, GI, maximum cord dose, mean cord dose, and MUs stratified by the number of fractions (one, three, and five) can be seen in Table 5. Trends consistent with the overall data set were observed. Elements plans had significantly better CI (*p* = 0.0017), GI (*p* = 0.0028), and maximum cord doses (*p* = 0.017) for single fraction plans. A trend toward lower mean cord dose (*p* = 0.076) was observed in single fraction plans. Average MUs for Elements plans were higher for single fraction plans but not statistically significant (*p* = 0.47).

**Table 5:** Summary of results stratified by the number of fractions with ranges of prescription doses used for each fractionation.

**Fractions**	**TPS**	**Prescription dose**	**CI**	**GI**	**Cord *D*_**max**_ (Gy)**	**Cord *D*_**mean**_ (Gy)**	**MU**
One (*n* = 10)	Clinical	12–24 Gy	1.25	4.21	12.50	4.36	5,816
	Elements		1.10	3.43	9.27	3.74	6,709
Three (*n* = 7)	Clinical	24–27 Gy	1.27	4.40	16.00	6.79	2,925
	Elements		1.10	3.64	13.00	5.14	3,212
Five (*n* = 3)	Clinical	30 Gy	1.23	3.78	18.70	9.46	1,914
	Elements		1.11	3.31	15.60	7.40	1,648

Three- and five-fraction plans showed the same trends for CI, GI, maximum cord dose, and mean cord dose; however, more data are needed to draw significant conclusions. Average MUs followed the overall data set trend for three fraction plans but were reversed for five fraction plans. More data are needed to draw significant conclusions for average MUs as well.

### Results Stratified by PTV Splitting

Dosimetric results in average absolute differences and average ratios of Elements values to current clinical values stratified by the amount of PTV splitting are summarized in Table 6. When the target is more complex and requires PTV splitting in Elements, the difference in CI and GI between Elements and current clinical plans is further accentuated (CI: *p* = 0.0091; GI: *p* = 0.0017) in favor of Elements. When target complexity increases, the difference in maximum and mean dose to the cord between Elements and current clinical plans decreases; however, values are still (cord *D*_max_: *p* = 0.14; cord *D*_mean_: *p* = 0.049) lower in the Elements plans.

**Table 6:** Dosimetric results stratified by the degree of PTV splitting.

	**PTV splitting**	**CI**	**GI**	**PTV *D*_**5%**_ (Gy)**	**Cord *D*_**max**_ (Gy)**	**Cord *D*_**mean**_ (Gy)**
None (*n* = 10)	Abs.	−0.14, 0.13	−0.51, 0.26	3.56, 1.98	−2.91, 0.78	−1.41, 1.14
	Dif. Ratio	0.90, 0.08	0.87, 0.06	1.14, 0.06	0.79, 0.07	0.72, 0.12
Once (*n* = 5)	Abs.	−0.11, 0.12	−0.68, 0.29	3.70, 0.71	−2.77, 0.99	−1.79, −1.11
	Dif. Ratio	0.92, 0.09	0.85, 0.05	1.15, 0.05	0.81, 0.05	0.76, 0.08
Twice (*n* = 5)	Abs.	−0.23, 0.25	−1.20, 1.10	1.40, 3.45	−3.77, 1.57	−1.80, 0.93
	Dif. Ratio	0.84, 0.14	0.78, 0.17	1.07, 0.17	0.75, 0.10	0.73, 0.09

*The first row of each PTV splitting category displays absolute differences between Elements and clinical plans (Elements–clinical), where a negative value indicates a lower Elements value. The second row in each PTV splitting category displays ratios (Elements values to clinical values) of the dosimetric parameters. Ratios >1 indicate a larger value in the Elements Spine SRS software. Ratios <1 indicate the opposite*.

For 8/10 cases where PTV splitting was used, Elements had more MUs and MFs, in contrast to only 4/10 cases where PTV splitting was not used. This indicates that PTV splitting has an effect on the plan's MU efficiency, which makes sense because it utilizes additional individual arcs for different parts of the PTV.

For Elements cases, calculation time increases as complexity of the target increases. The average time to complete the pencil beam and MC calculations for cases where PTV splitting was not used (2 arcs) was 10.2 ± 2.3 min. The overall average calculation time for the 20 cases was 27.9 min. Because actual computation time will vary largely by computer specifications and version of software, it is more useful to assume that compared to no target splitting, computation time will increase when PTV splitting occurs. This may influence an institution's preference toward how much splitting flexibility to allow the software because this is a controllable parameter. Calculation time also depends on grid calculation size and statistical uncertainty requested of the MC algorithm, which are both user defined.

## Discussion

### General

The maximum PTV dose for Elements plans was significantly higher compared to clinical plans. This could also affect the dose coverage and low-dose spread, as we know that more heterogeneous dose inside PTV means easier way to achieve sharper dose fall off and less low-dose spread. However, there is an additional dose homogeneity constraint that can be utilized to control the hot spot in Element. It was not used for the purposes of this study, and the hot spot in the target was acceptable for all cases.

Trends were consistent among all prescription/fractionation schemes. Average total MUs for Elements were larger than in current clinical plans for one- and three-fraction plans and lower for five-fraction plans (Table 5).

The automated planning available in Elements enables planners to create a quick treatment plan with consistent interplanner and intraplanner quality. Planner variability may have influenced the quality of current clinical plans in comparison to Elements plans; however, all plans were clinically acceptable, and this emphasizes the benefit of reproducible plan quality in automated planning. There are other automated planning tools, such as Rapidplan, which can be used as option for achieving better and more consistent plans.

The Elements MC step provides a highly accurate optimization and calculation with a pencil beam starting point. The fact that the TPS not only calculates the dose with MC, but also utilizes a final MC optimization, allows fine tweaking of the dose distribution to increase coverage and critical structure sparing in scenarios of highly heterogeneous media or loss of lateral charged particle equilibrium. Results from section B, *Monte Carlo* vs. *Pencil Beam*, shows that benefits exist when using MC even for cases where there is not a large amount of heterogeneity.

Despite only utilizing spinal cord constraints in the Elements plan optimizations, all clinically specified critical structure objectives used at our clinic for the approved clinical plans were met. This may mean that tighter tolerances can be achievable with the Elements software or that the constraints were simply easily met in both planning systems. Further investigation is warranted before coming to any conclusions; however, this may mean that critical structure objectives for spine SRS and SBRT can be tightened at our institution.

### Monte Carlo vs. Pencil Beam

To assess changes in plan quality and the impact of MC after the pencil beam calculations, the dosimetry from both algorithms for 20 cases were compared. To do so, the exact pencil beam–calculated plan was recalculated using the MC algorithm, and dosimetry was compared between the two. Target coverage normalization was not performed after the recalculation. Pencil beam calculations were recalculated with the MC algorithm to compare changes in heterogeneous situations. It is important to note that the pencil beam plans recalculated with MC were not further optimized with MC and not renormalized to 95% coverage. They are reported as is with a direct MC recalculation.

When comparing results, two groups were analyzed separately. Group 1 contains eight cases in T spine region where large heterogeneity was presented (lung), and group 2 has 12 cases in C-spine, L-spine, and S-spine region with more homogeneous tissues. Coverage for the MC plans decreased from 95% in the pencil beam plans to 91.9 ± 1.9% and 93.9 ± 1.1% for groups 1 and 2, respectively, which is as expected because of the larger heterogeneity presented in group 1 cases. Conformity index was lower after recalculating for most plans, which is due to differences in target coverage resulting from not renormalizing the recalculated plans. Cord maximum doses in MC plans were 3.4 ± 4.7% lower for group 1 cases, whereas they were 0.3 ± 3.6% higher for group 2 cases.

It can be seen from the results that for T-spine cases where large heterogeneous tissue (lung) was presented, MC algorithm is better to be used to avoid coverage loss and more accurate cord dose (~3% difference for both). For other spine regions, users may decide to use pencil beam only to save planning time.

### Planning Comparison Limitations

The clinical plans were generated prior to Elements Spine SRS package available at our institution, and they were not replanned and reoptimized. The purpose of this study is not to compare Elements vs. another TPS. These available clinical plans were used only as measures to evaluate Elements plans because they were clinically accepted (does not mean they were best optimized). If our purpose is to compare different planning systems' capability of coming up with optimized plans, the Eclipse optimizer could definitely be pushed harder to achieve better plans. Also, newer versions of Eclipse TPS with newer optimizer should be used for the comparison. Unfortunately, the newer version and the newer optimization algorithms have not been installed at our institution yet. It is our future goal to study once we have it installed.

Elements Spine SRS is designed to be a dedicated system for spine SRS/SBRT planning and hence would not compare to other generic TPS in terms of flexibility, such as to modify calculated plans, to set up arc geometries, and to modify patient specific OAR constraints. There are limited functionalities in contouring modules compared to other standard full-scale TPS, too.

The reasons for the dosimetric differences we observed in current study could be attributed to the following: (1) different arc geometry was used, especially for the complicated cases, where Elements automatically uses the so-called “PTV splitting” technique, so that different arcs are used to concentrate on different part of the PTV. For these cases, it used four to six full arcs so that the plan inherently has more optimization space compared to the clinical plans, whereas only two full arcs were used clinically; (2) Elements plans have more MUs and MFs and thus will have longer delivery time. Therefore, we can say that comparing to our clinical plan it achieved better dosimetry at the expense of more arcs, more MUs, and longer delivery time.

## Conclusions

The Elements Spine SRS plans were superior to current clinical plans in achieving high conformity, decreasing GI, and sparing dose to the cord. Increase in number of arcs, MUs, or MFs was noted. The automation in Elements was shown to consistently generate plans with quality as good as or better than our current clinical plans. Furthermore, automation provided by Elements may allow clinicians to spend more time on other aspects of a patient's care rather than treatment planning. Whereas, other algorithms can also utilize templates to reduce manual efforts, the Elements VMAT implementation for spine is specific to creating a steep dose gradient toward the spinal cord and may thus help to reduce the probability of one of the primary mechanisms of local failure after spine SRS/SBRT. PTV splitting in Elements is helpful to successfully shape dose around complex targets.

## Data Availability Statement

All datasets generated for this study are included in the article/supplementary material.

## Ethics Statement

The study was approved by the office of human research, institutional review board (IRB) of Thomas Jefferson University. The IRB has granted a waiver of informed consent.

## Author Contributions

MT performed the data collection, planning, and drafting manuscript. AL performed planning and assisting with manuscript and figure generation. YY, WS, and HL developed the methods and setup the planning parameters for comparison. HL addressed both reviewers' questions and comments, and finalized the manuscript.

### Conflict of Interest

Thomas Jefferson University has a research agreement with Brainlab AG for evaluation of Elements Spine SRS technology, for which HL and WS are the principal investigators. The funder was not involved in the study design, data collection, analysis, and interpretation, the writing of this article or the decision to submit it for publication. The remaining authors declare that the research was conducted in the absence of any commercial or financial relationships that could be construed as a potential conflict of interest.
